# Early nutritional intake influences the serum levels of nerve growth factor (NGF) and brain-derived neurotrophic factor in preterm newborns

**DOI:** 10.3389/fneur.2022.988101

**Published:** 2022-10-17

**Authors:** Maria Chiara De Nardo, Carla Petrella, Maria Di Chiara, Chiara Di Mario, Giorgia Deli, Elisa Travaglia, Laura Baldini, Alessia Russo, Pasquale Parisi, Marco Fiore, Gianluca Terrin

**Affiliations:** ^1^Department of Maternal and Child Health Policlinico Umberto I, Sapienza University, Rome, Italy; ^2^Institute of Biochemistry and Cell Biology (IBB) of the National Research Council (CNR), Rome, Italy; ^3^Department of Pediatrics, Mental Health and Sense Organs (NESMOS), Faculty of Medicine and Psychology, c/o Sant'Andrea Hospital, Sapienza University, Rome, Italy; ^4^Department of Neuroscience, Mental Health and Sense Organs (NESMOS), Faculty of Medicine and Psychology, c/o Sant'Andrea Hospital, Sapienza University, Rome, Italy

**Keywords:** enteral nutrition, parenteral nutrition, nerve growth factor (NGF), brain-derived neurotrophic factor (BDNF), preterm neonates

## Abstract

**Introduction:**

Parenteral nutrition (PN) may have detrimental effects on neurodevelopment in preterm newborns. Moreover, enteral nutrition (EN) seems to be protective. To understand the mechanisms of how neurological development can be influenced by the route of administration of nutritional intake, we investigated the relationship between the serum levels of the nerve growth factor (NGF) and brain-derived neurotrophic factor (BDNF) and nutritional intake received in early life by preterm newborns.

**Materials and methods:**

Specimens of blood were obtained at 28 days of life (DOL) for NGF/BDNF determination in neonates <32 weeks of gestation and/or with birth weight <1,500 g, consecutively observed in the neonatal intensive care unit. We analyzed the relation between amino acid content and energy intake and NGF/BDNF measurements at 28 DOL. PN protein intake was referred to as the total amounts of amino acid intake received daily.

**Results:**

We enrolled 20 newborns (gestational age 30.45 ± 1.76 weeks, birth weight 1,340 ± 352.63 g). Serum NGF value at 28 DOL was positively correlated with enteral protein and energy intake (*r* = 0.767; *r* = 0.746, *p* < 0.001), whereas, negatively correlated with parenteral amino acid and energy intake (*r* = −0.652, *p* < 0.001; *r* = −0.466, *p* < 0.05). Similar significant correlations were described between BDNF level at 28 DOL and enteral energy intake (*r* = 0.493, *p* < 0.05). Multivariate regression analysis showed that NGF level at 28 DOL depends on enteral protein and energy intake administrated in the 1st week of life.

**Conclusion:**

Neurotrophin values varied according to the route of nutrition administration in preterm newborns. NGF/BDNF serum levels are influenced positively and negatively by EN and PN, respectively.

## Introduction

Controversies exist regarding the effects of high recommended nutritional intake in early life and cerebral development. Several studies focused on the relationship between enhanced nutritional protocols and neurological outcomes with uneven results. It has been reported a positive correlation between higher energy and protein intakes and better cerebral maturation, measured by magnetic resonance imaging (MRI) (1). We recently demonstrated that elevated energy and amino acid intakes have different effects whether administered by enteral (EN) or parenteral (PN) nutrition (2). Early-enhanced PN, in particular, had a negative effect on cerebral growth (i.e., linear measurement of cerebral structures and major diameters) as assessed by cranial ultrasound in two observational studies, whereas similar intake administered *via* EN appeared to be positively correlated with cerebral growth (3, 4). In addition, a recent study comparing two PN protocols (standard vs. early-enhanced) demonstrated that an increased intake of macronutrients early in life results in lower motor scores and socioemotional competence performance at 24 months of life (5). Some authors studied enteral nutritional intakes and found a positive correlation between protein and energy intakes and caudate nuclei growth as well as intelligence quotient (IQ) later in life (6). Other RCTs did not find any difference in the control or intervention group in terms of neurodevelopment delay (7, 8). Understanding the mechanisms of how the route of administration of macronutrient nutritional intakes influences neurological development could be useful in clinical practice in determining the optimal nutritional strategy for preterm babies. Neurotrophic factors (NFs) are secretory proteins involved in the promotion of neurons' development, maintenance and survival and synaptic remodeling in central and peripheral nervous tissue (9–11). The NFs include primarily the nerve growth factor (NGF), the first to be discovered, and the brain-derived neurotrophic factor (BDNF) (12–14). Besides, both NGF and BDNF play key roles in regulating physiological functions in non-neuronal populations (15–17). They were also shown to stimulate the axonal sprouting formation and promote axonal functional reconnection, which was correlated with functional recovery after ischemia stroke in the adult population (18). It has been recently demonstrated that preterm newborns compared to term infants have a lower level of NGF in maternal and cord blood plasma (19). A systematic review and meta-analysis indicated that lower levels of BDNF in both cord and peripheral blood may be associated with preterm birth. However, the analysis of the studies revealed a significant heterogeneity between the specimens used for NFs analysis. In particular, samples for NFs determination were collected from peripheral blood of newborns, umbilical cord blood, and cerebrospinal fluid and placental tissue (9). Some studies reported relevant clinical correlations between NGF levels and neurodevelopmental outcomes among the neonatal population (13). Krey et al. demonstrated that NFs' levels influence motor and cognitive function (12). In observational studies, it has been demonstrated that NGF has a positive correlation with the Bayley score at 24 months of life and brain volumes, implying that this neurotrophin can be used to identify infants at high risk of NDV (20, 21). It has demonstrated the presence of NFs in breast milk and higher levels of NGF in breastfed infants, born with small gestational age weight (22–24). However, no study investigated the relationship between nutrition and NGF and BDNF values. We investigated the relationship between the serum levels of the neurotrophins (NGF/BDNF) and nutritional intake received in early life by preterm newborns.

## Materials and methods

### Study design and population

We included all newborns with gestational age (GA) <32 weeks and/or body birth weight (BW) <1,500 g, consecutively admitted to the NICU of Policlinico Umberto I, La Sapienza University of Rome, between February 2021 and August 2021. We excluded infants with major congenital malformations, inborn errors of metabolism, congenital infections, intraventricular hemorrhage (IVH) stage ≥ 3, death, or transfer to another hospital before 72 h of life (25–28).

We also included 10 full-term newborns with adequate body birth weight for gestational age who were born spontaneously after an uneventful pregnancy as controls.

### Collection data

Prenatal, perinatal, and postnatal information were prospectively collected for each patient in specific data forms. In particular, GA, BW, gender, type of delivery, twin pregnancy, antenatal steroid administration, Apgar score at 1st and 5th min after birth, pH on cord blood at birth, body temperature at the 1st h of life, Clinical Risk Index for Babies II score (CRIB II) death, and need of invasive mechanical ventilation were recorded (29). Diagnosis of the major morbidities associated with prematurity, such as necrotizing enterocolitis (NEC, Bell stage ≥ 2), bronchopulmonary dysplasia (BPD), intraventricular hemorrhage (IVH), periventricular leukomalacia (PVL), retinopathy of prematurity (ROP), and sepsis proven by positive cultures were performed according to the standard criteria and recorded in the reporting form, as previously described (30, 31). Data on daily enteral and parenteral nutritional intake were collected. On the basis of the duration of PN, we classified preterm enrolled newborns in group A, including preterm babies receiving PN for at least 14 days, and group B including subjects receiving PN < 14 days. We measured the head circumference of the newborns with a tape measure. We wrapped the tape, holding it above the eyebrows and the ears and the occipital prominence at the back of the skull, around the widest possible circumference of the head.

### Nutritional protocol

Enteral nutrition (EN) was commenced as soon as possible after birth in a stable newborn. Minimal enteral feeding was started within 24–48 h after birth at 10–20 ml/kg/day. The amount was increased by 20–30 ml/kg every day if EN was tolerated. Maternal milk (MM) without fortifications if available has been given fresh. Whether MM was not available or sufficient, a formula specifically formulated for preterm newborns and routinely given in our NICU was used. Donor breast milk during the study period was not available. When signs or symptoms of feeding intolerance like emesis, vomiting, severe abdominal distension associated with ileus with visible intestinal loops, blood in the stools, or systemic disorders (i.e., apnoea, bradycardia, inadequate perfusion, and hemodynamic instabilities) were observed, the EN was withheld for at least 24 h (32). Macronutrient contents of formula and parenteral solutions were calculated based on the published manufacturers' recordings. Parenteral nutrition (PN) was administered at birth to maintain adequate fluid, electrolyte, and nutrient intakes until exclusive enteral feeding (120 kcal/kg/day) was achieved. The overall fluid intake administered with enteral and PN started with 80 ml/kg/day and slowly increased by 10–20 ml/kg/day until reaching150 ml/ kg/day. In PN, we administered 2,5 g of amino acids (TrophAmine^®^ 6% Braun Medical Inc. Irvine, USA) in the 1st days of life (DOL), then we increased amino acid intake up to 3,2 g/Kg/day, with 25 kcal per 1 g of amino acid. Glucose intake (dextrose injection 10%, Fresenius Kabi, USA) was started at 6 g/kg/day and increased up to 13 g/Kg/day. Lipid (Smoflipid^®^, Fresenius Kabi, USA) intake was started at 1 g/kg/day and increased up to 3.5 g/kg/day. Preterm HM was assumed to contain 65 Kcal/100 ml (1.5 g of protein/100 ml, 3.5 g of fat/100 ml, 6.9 g of carbohydrate/100 ml). Macronutrient contents of the formula (Pre-Nidina Nestlè^®^: proteins 2.9 g/dL, lipids 4.0 g/dL, energy 8.1 g/dL) were calculated based on the published manufacturer's labels. Total energy intake was calculated based on the cumulative amount of parental and EN in kcal/kg over the early 7 days. Target dose refers to enteral plus PN; thus, we adjusted intake from PN according to the amount of EN tolerated.

### NGF and BDNF protein assays

Specimens of blood (0.5 ml) were obtained from preterm newborns at 0 and 28 days of life. We also collected a specimen of blood from full-term control babies at day 0 of life. Samples at day 0 were collected from umbilical cord blood. The length of clotting time was 1 h at room temperature (33, 34). The serum separated from the blood was stored at −20°C until NGF/BDNF determination. NGF (Cat. No. DY256) and BDNF (Cat. No. DY248) were measured using sandwich enzyme-linked immunosorbent assay (ELISA) kits (R&D Systems, Minneapolis, MN, USA), according to the protocols provided by the manufacturer and also according to methods previously described (35). Serum samples were diluted 2- and 100-fold with PBS for the detection of NGF and BDNF, respectively. The colorimetric reaction product was measured at 450 nm using a microplate reader (Neo Biotech Microplate Reader, Italy). Data are represented as ng/ml (BDNF) or pg/ml (NGF) and all assays were performed in duplicate which was averaged for statistical comparison.

### Statistical analysis

Data analysis was performed using IBM the Statistical Package for the Social Sciences Statistics version 22.0 (SPSS Inc, Chicago, IL). We checked for normality using the Shapiro–Wilk test. The mean and standard deviation or median and interquartile range summarized continuous variables. We compared categorical variables using the chi-square test and paired and unpaired variables by *t*-test, ANOVA, or Mann–Whitney *U*-test. Nutritional intake was correlated with the serum level of NGF and BDNF at 28 days of life (T1), by Wilcoxon rank sum tests and by Pearson's correlation. Multivariate regression analysis was performed to study the possible influence of confounding variables (i.e., gestational age, pH on cord blood, CRIB2 score, parenteral and enteral energy intake, parenteral amino acid, and enteral protein intake in the 1st week of life) on the serum level of neurotrophins at 28 days of life. The level of significance for all statistical tests was two-sided (*p* < 0.05).

### Ethics

The study was conducted in conformity with the World Medical Association Declaration of Helsinki for medical research involving human subjects. This article reports a part of the results of the study protocol that was approved by the Ethics Committee of Policlinico Umberto I, University La Sapienza of Rome (with number 5089). We asked parents for consent, and we collected anonymized collected in the database.

## Results

In Table 1, we reported the baseline demographic and clinical features of 20 preterm enrolled newborns. Ten full-term controls showed a mean body birth weight of 3,350 ± 352.2 gr of and a mean gestational age of 39.4 ± 1.5. All full-term newborns received exclusively breast milk. Eleven out of 20 preterm babies had EN withheld for at least 24 h (10 among subjects that received more than 14 days of PN and one among babies that received PN <14 days).

**Table 1:** Clinical characteristics of the study population.

***N. 20***	
Antenatal steroids^a^, *N. (%)*	11 (55)
Gestational age, *weeks*	30.45 ± 1.76
Birth weight, *g*	1,340 ± 352.63
Cesarean section, *N. (%)*	17 (85)
Male sex, *N. (%)*	9 (45)
Twins, *N. (%)*	6 (30)
1-min Apgar score	5.8 ± 2.50
5-min Apgar score	7.9 ± 1.71
pH at birth	7.23 ± 0.10
Base excess on cord blood, *mmol/L*	−6.7 ± 5.76
Temperature at the 1st hour, *°C*	36.1 ± 0.5
CRIB II score^b^	6.0 ± 3.49
Mortality, *N. (%)*	0 (0)
NEC, *N. (%)*	3 (15)
IVH, *N. (%)*	0 (0)
PLV, *N. (%)*	0 (0)
Sepsis proven by positive cultures, *N (%)*	5 (25)
ROP, *N. (%)*	4 (20)
BPD, *N. (%)*	1 (5)
Anemia of prematurity, *N. (%)*	5 (25)
Invasive mechanical ventilation, *N. (%)*	4 (20)
Full enteral feeding, *days of life*	23.3 ± 19.3
Start of enteral nutrition, *days of life*	1.84 ± 1.64
Exclusively or partial HM at 14 days, *N (%)*	15 (75)
Only formula milk at 14 days, *N (%)*	3 (15)
Parenteral nutrition at 14 days, *N (%)*	2 (10)
Duration of parenteral nutrition, *days*	20.8 ± 19.8

^a^Intramuscular steroids cycle in two doses of 12 mg over a 24-h period.

^b^CRIB II, clinical risk index for babies, without temperature measures; NEC, necrotizing enterocolitis; IVH, intraventricular hemorrhage; PLV, periventricular leukomalacia; ROP, retinopathy of prematurity; PDA, patent ductus arteriosus; BPD, bronchopulmonary dysplasia; HM, human milk.

Data were expressed as mean ± standard deviation, when not specified.

The serum specimens from 19 newborns were included in the neurotrophins analysis as one sample was lost because of the small sample volume.

**Figure 1:**
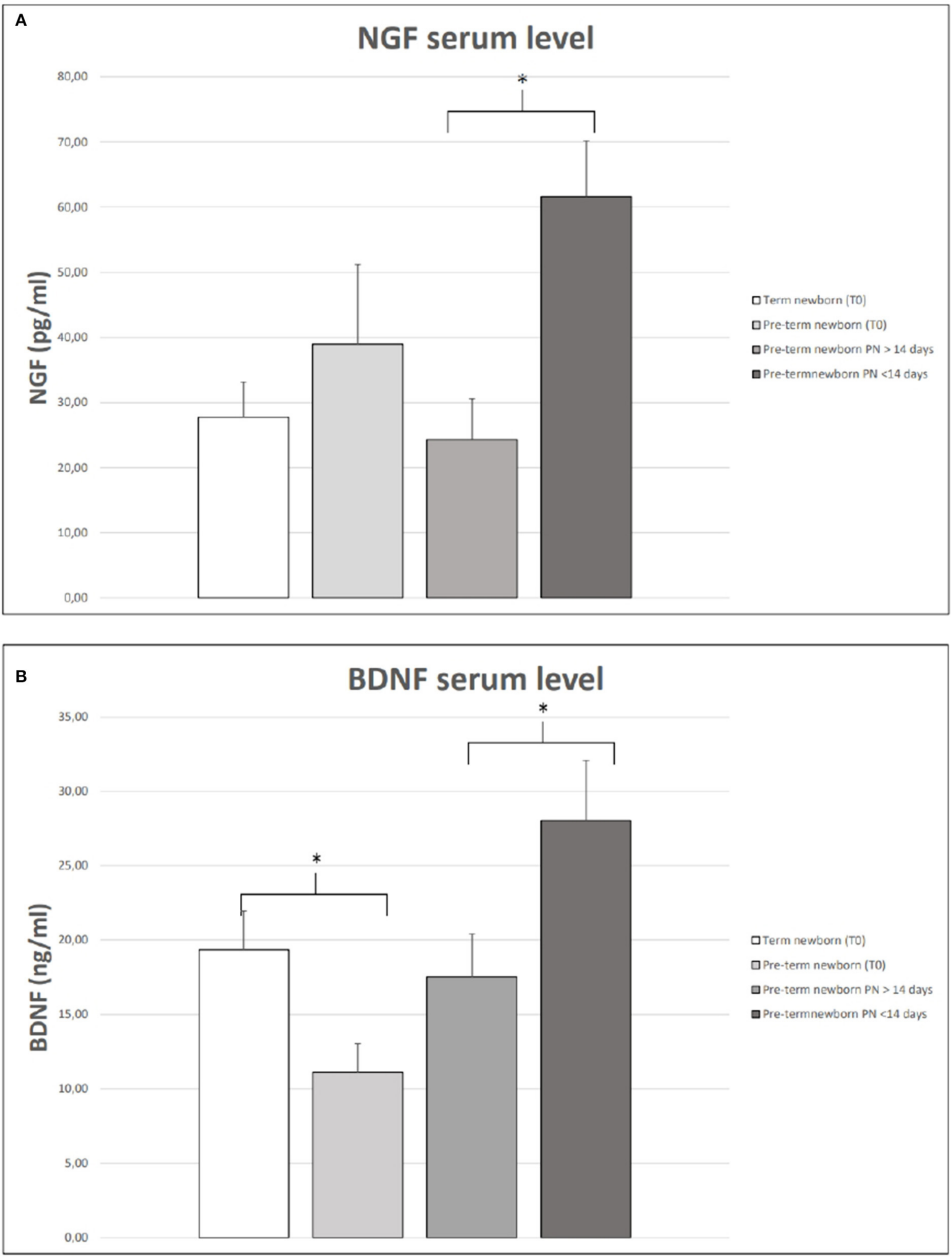
NGF **(A)** and BDNF **(B)** serum levels in term and preterm neonates at birth (T0). Serum NGF **(A)** and BDNF **(B)** at 28 days of life in preterm babies receiving parenteral nutrition (PN) for more or less 14 days of life. The vertical lines extending from each box represent the standard error. The asterisks indicate significant differences between groups (*p < 0.05).

We observed that the serum levels of BDNF were significantly higher in full-term newborns than in preterm newborns (Figure 1). In Figure 1, we also showed that, among preterm newborns, the levels of NFs were lower in preterm babies receiving PN for more than 14 days of life. We found that the levels of BDNF and NGF at birth and 28 days of life were similar among female and male babies. We also observed no difference in NFs serum levels in newborns who received antenatal steroids compared to those who did not receive antenatal steroids.

**Figure 2:**
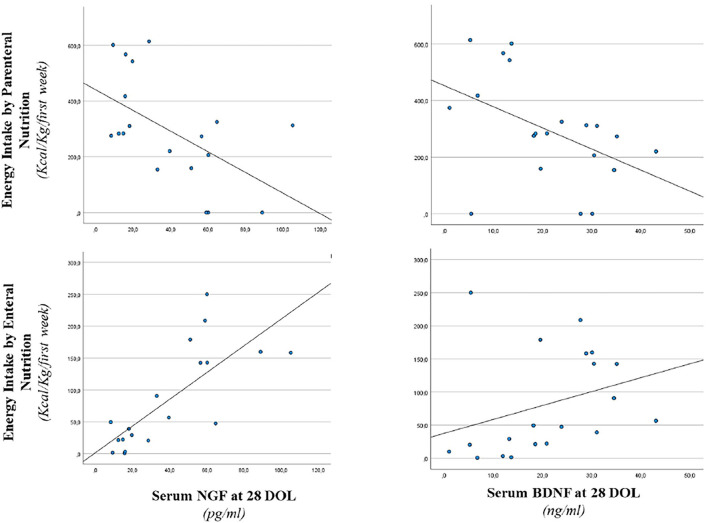
Correlation between serum NGF and BDNF level at 28 days of life and energy intake. Enteral nutrition: serum NGF (*r* = 0.746, *p* < 0.001) and serum BDNF (*r* = 0.493, *p* < 0.05). Parenteral nutrition: serum NGF (*r* = −0.466, *p* < 0.05) and serum BDNF (*r* = −0.530, *p* < 0.05).

In Figure 2, we reported a correlation between serum NGF and BDNF levels at 28 days of life and energy intake in the 1st week of life. In particular, we observed a positive correlation between enteral energy intake and serum NGF (*r* = 0.746, *p* < 0.001) and a negative correlation between parenteral energy intake and serum NGF level (*r* = −0.466, *p* < 0.05). Similar significant correlations were described between BDNF level at 28 days of life and enteral energy intake (*r* = 0.493, *p* < 0.05). A negative correlation was noticed between BDNF value and parenteral energy intake (*r* = −0.530, *p* < 0.05) as shown in Figure 2.

**Figure 3:**
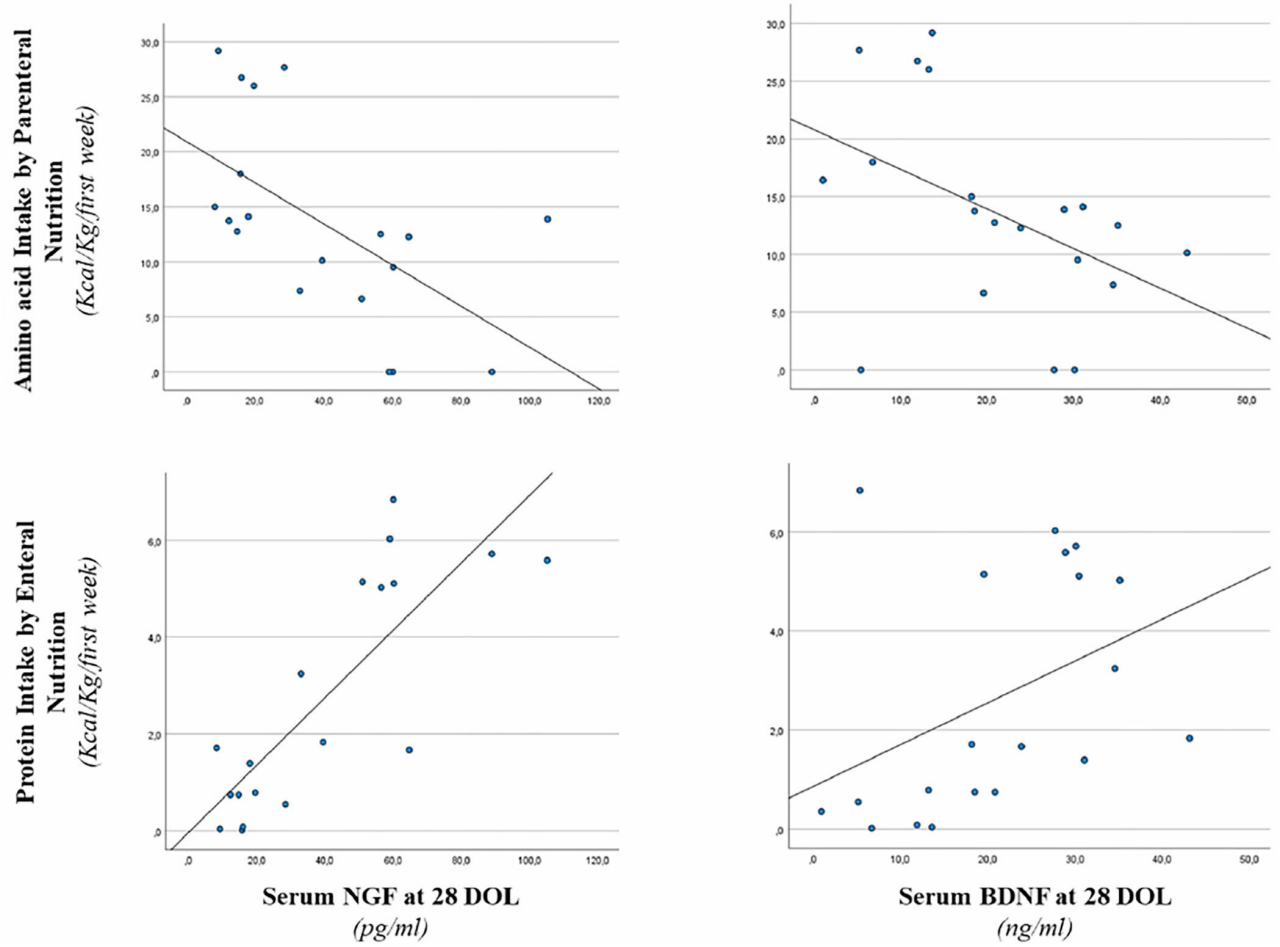
Correlation between serum NGF and BDNF level at 28 days of life, enteral protein intake, and parenteral amino acid intake. Enteral nutrition: serum NGF (*r* = 0.767, *p* < 0.001) and serum BDNF (*r* = 0.544, *p* < 0.05). Parenteral nutrition: serum NGF (*r* = −0.652, *p* < 0.001) and serum BDNF (*r* = −0.529, *p* < 0.05).

Correlations between serum NGF and BDNF levels at 28 days of life and early protein intake are shown in Figure 3. Serum NGF value at 28 days of life was positively correlated with EN protein intake (*r* = 0.767, *p* < 0.001), whereas, negatively correlated with PN amino-acid intake (*r* = −0.652, *p* < 0.001). We also found a positive correlation between EN protein intake and serum BDNF (*r* = 0.544, *p* < 0.05). Negative correlation between parenteral energy intake and serum BDNF level (*r* = −0.529, *p* < 0.05) was also observed (Figure 3).

Results observed in univariate analysis were corrected by confounding variables. Multivariate regression analysis showed that NGF levels at 28 days of life depend on protein and energy intake administrated by enteral nutrition in the 1st week of life (Table 2). No relations were observed between BDNF and gestational age, pH on cord blood, CRIB2 score, parenteral and enteral energy intake, parenteral amino acid, and enteral protein intake in the 1st week of life (Supplementary Table S1).

**Table 2:** Multivariate analysis of covariates influencing NGF serum level at 28 days of life in preterm newborns.

**Dependent variables**	**NGF serum level°**	**B**	**S.E**.	**β**	***p*-value**	**95% CI for OD**	
						**Lower**	**Upper**
Covariates (model 1)	Gestational age	3.259	4.289	0.280	0.469	−6.631	13.149
	PH on cord blood	37.007	62.067	0.119	0.567	−106.119	180.133
	CRIB2 score	−2.865	3.345	−0.414	0.417	−10.578	4.848
	PN energy intake 0–7 DOL, *kcal/Kg/week*	0.069	0.046	0.595	0.171	−0.037	0.175
	EN energy intake 0–7 DOL, *kcal/Kg/week*	0.223	0.068	0.838	0.012*	0.065	0.381
Covariates (model 2)	Gestational age	2.995	3.548	0.257	0.423	−5.188	11.177
	PH on cord blood	35.358	51.482	0.114	0.512	−83.361	154.076
	CRIB2 score	−3.178	2.939	−0.459	0.311	−9.956	3.600
	PN amino acid intake 0–7 DOL, *g/Kg/week*	1.798	0.990	0.652	0.107	−0.484	4.080
	EN protein intake 0–7 DOL, *g/Kg/week*	7.362	1.718	0.926	0.003*	3.399	11.324

CRIB II, clinical risk index for babies; EN, enteral nutrition; PN, parenteral nutrition.

°Measured at 28 days of life.

^*^p < 0.05.

## Discussion

We observed that nutritional intake given early in life influences serum levels of NGF and BDNF in preterm newborns. We demonstrated that the duration of PN may influence the level of serum NFs. Furthermore, we observed that neurotrophin serum levels varied according to the route of administration of macronutrients: enteral or parenteral. Specifically, serum NGF and BDNF are positively influenced by the administration of energy and protein intake through the enteral route, whereas NFs are adversely affected by parenteral energy and amino acid intake. Results corrected for confounding variables confirmed that early nutritional intake significantly influences serum NGF value. Previous studies on neurotrophins are focused mainly on the comparison of these biomarkers between preterm and term infants. For the first time, we demonstrated that nutritional intake may have different effects on NFs levels, if given by enteral or parenteral route, confirming our previous evidence about the consequences of the route of nutrient administration on cerebral growth (36). In a prospective study, Madhavi et al. described a reduced level of NGF in maternal and cord blood of preterm babies differently to term infants (19). No information regarding nutrition was explored by the authors. Previous studies exploring the effects of antenatal steroids on neurotrophic factor levels in preterm newborns are controversial. Some authors found that NGF and BDNF levels were unaffected by antenatal steroid exposure whereas antenatal steroids reduced cord blood neurotrophin-3 (NT-3) (37). This result is in contrast to another study, which reported a significantly higher level of BDNF in samples from subjects who received a complete course of antenatal steroids compared with those with no antenatal steroids (38). In our study, we found that levels of BDNF and NGF at birth and at 28 days of life were similar among female and male babies. We also observed no difference in NFs at birth and at 28 days of life in newborns who received antenatal steroids compared to those who did not receive antenatal steroids. However, considering the small number of neonates included in the study, our results could not exclude that NFs' levels could vary with sex or antenatal steroids exposure. Further studies are advocated to address this aspect. Some studies reported relevant clinical correlations between serum NGF levels and neurodevelopmental outcomes among the neonatal population, suggesting that these neurotrophins should be further explored as a biomarker of neuronal repair (20, 39). Similar conclusions were drawn by Aisa et al., which correlated urinary NGF measured at 30–40 days of the postnatal period with brain growth, assessed at the same time point, and with the neurodevelopment outcome at 2 years of age evaluated by the Griffiths-II test (21). Similar investigations were conducted also by Massaro et al. which correlated serum BDNF value with NDV outcome in newborns with hypoxic-ischemic encephalopathy (36). Nevertheless, in all these studies, the authors did not consider the effects of nutrition on neurotrophins production. Therefore, it is not possible to exclude that the correlation between serum NFs and different neurodevelopment outcomes among preterm newborns could be influenced by nutritional intake (4). Only a few studies have focused on the relation between neurotrophic factors and nutrition. It has been described that BDNF is present in human milk and levels of NGF are higher in breastfed infants, born with small for gestational age weight (22–24). However, no studies have yet explored the differences between NGF and BDNF values according to enteral and parenteral nutrition.

We hypothesized that some mechanisms may support the observed effects. It has been recently suggested that the brain–gut axis is a communication system that integrates neural, hormonal, and immunological signaling between the gut and the brain. The interaction with the brain–gut axis may represent a potential route to influence brain function and development. However, the exact mechanisms by which the gut communicates with the brain have not been elucidated yet (40). We theorized that the trophic stimulation of intestinal mucosa by enteral feeding could promote NFs secretion in peripheral tissue (41). It is well-described that starvation could affect neurodevelopment in infants born preterm (42). It is conceivable that NFs released through the pathway of brain–gut axis could have the function of neuronal development. Moreover, a lower concentration of BDNF, mainly expressed in the central nervous system, has been reported in peripheral tissue in comparison with other NFs (12). This biological characteristic may explain different results between NGF and BDNF observed in our study.

### Limitation of the study

Despite being interesting, the results of this study should be interpreted by taking into account specific limitations. First, the association between early nutritional intake and NFs level at 28 days of life may be related to the effects of chance (random error), bias, or confounding factors. We verified that effects on NFs levels persisted even after correcting for confounding variables. We also included 10 full-term newborns with adequate body birth weight for gestational age who were born spontaneously after an uneventful pregnancy as controls. To limit associated bias, the researchers involved in NFs measurements were unaware of the nutritional intake and other clinical information. To limit methodological bias, we explicitly stated the length of clotting time of serum samples as we know that this is a key methodological criterion for a correct serum BDNF dosage (33, 34). The study enrolled a small number of patients; however, our data highlight marked differences between subjects receiving prolonged NP and those that reach full enteral feeding within 14 days of life (*post-hoc* power at 95.4%). Finally, no data on long-term neurodevelopment were analyzed in this study. Thus, it is not possible to establish if the results observed at 28 days of life on NFs level may be a reliable biomarker of long-term neurodevelopment.

## Conclusion

In conclusion, we observed that NGF and BDFN are influenced by the route of nutrition administration in preterm newborns. Given this result, a future scenario is represented by the possibility of considering NFs as markers of nutritional tolerance and target for new potential nutritional strategies and as a tool to predict growth and brain development in preterm newborns. However, considering the limitation of our study, further studies are advocated to confirm our observations. Further research should aim to clarify molecular mechanisms involved in NFs levels related to the route of nutrition in the early life of preterm neonates and to explore NDV outcomes resulting from altered levels of NFs among preterm infants.

## Data availability statement

The raw data supporting the conclusions of this article will be made available by the authors, without undue reservation.

## Ethics statement

The study was conducted in conformity with World Medical Association Declaration of Helsinki for medical research involving human subjects. This paper reports a part of the results of the study protocol that was approved by Ethics Committee of Policlinico Umberto I, University La Sapienza of Rome (with number 5089). We asked parents for consent, and we collected anonymized collected in the database. Written informed consent to participate in this study was provided by the participants' legal guardian/next of kin.

## Author contributions

MDe and GT contributed to the design of the research. CD, GD, LB, CP, and MDe contributed to the acquisition and analysis of the data. MDi, AR, and ET contributed to the analysis of the data. MF, CP, PP, and GT contributed to the acquisition, analysis, and interpretation of the data. All authors drafted the manuscript, critically revised the manuscript, agree to be fully accountable for ensuring the integrity and accuracy of the work, and read and approved the final manuscript.

## Conflict of interest

The authors declare that the research was conducted in the absence of any commercial or financial relationships that could be construed as a potential conflict of interest.

## Publisher's note

All claims expressed in this article are solely those of the authors and do not necessarily represent those of their affiliated organizations, or those of the publisher, the editors and the reviewers. Any product that may be evaluated in this article, or claim that may be made by its manufacturer, is not guaranteed or endorsed by the publisher.
