# Supplementary material for: Early nutritional intake influences the serum levels of nerve growth factor (NGF) and brain-derived neurotrophic factor in preterm newborns
Source: Front Neurol. 2022 Oct 17;13:988101. doi: 10.3389/fneur.2022.988101 (PMC9620475; doi:10.3389/fneur.2022.988101)
Supplement: Supplementary file 1 [file Table_1.docx]

Supplementary Material

**Supplementary Table 1.** Multivariate analysis of covariates influencing BDNF serum level at 28 days of life in preterm newborns

| *Dependent variables* | **BDNF serum level°** | **B** | **S.E.** | **β** | ***p-value*** | **95% CI for OD** | |
| --- | --- | --- | --- | --- | --- | --- | --- |
|  |  |  |  |  |  | **Lower** | **Upper** |
| **Covariates (Model 1)** | **Gestational Age** | -1.143 | 2.925 | -0.239 | 0.705 | -7.760 | 5.475 |
|  | **PH on cord blood** | -26.534 | 42.335 | -0.208 | 0.546 | -122.302 | 69.234 |
|  | **CRIB2score** | -1.192 | 2.281 | -0.419 | 0.614 | -6.353 | 3.659 |
|  | **PN energy intake 0–7 DOL, *kcal/Kg/week*** | -0.014 | 0.031 | -0.286 | 0.674 | -0.085 | 0.057 |
|  | **EN energy intake 0–7 DOL, *kcal/Kg/week*** | 0.006 | 0.047 | 0.058 | 0.895 | -0.099 | 0.112 |
| **Covariates (Model 2)** | **Gestational Age** | -1.430 | 2.870 | -0.298 | 0.630 | -7.922 | 5.062 |
|  | **PH on cord blood** | -31.170 | 41.638 | -0.244 | 0.473 | -125.361 | 63.020 |
|  | **CRIB2score** | -1.714 | 2.377 | -0.603 | 0.489 | -7.092 | 3.664 |
|  | **PN amino-acid Intake 0–7 DOL, *g/Kg/week*** | -0.001 | 0.800 | -0.001 | 0.999 | -1.812 | 1.810 |
|  | **EN protein Intake 0–7 DOL, *g/Kg/week*** | 0.981 | 1.390 | 0.300 | 0.498 | -2.162 | 4.125 |

**Notes**. CRIB II, Clinical Risk Index for Babies; EN = Enteral Nutrition; PN = Parenteral Nutrition; ° Measured at 28 days of life; ***** p
